# Assessment of Understandability and Actionability of YouTube Videos on Hemolytic Disease of the Newborn

**DOI:** 10.7759/cureus.33724

**Published:** 2023-01-12

**Authors:** Iruvaram Sudhir Chaitanya Kumar, Anila Mani, TVN Sriranjitha, I Muni Srikanth, KV Aswathy, Shesh Kumar Bhakta, Prudhvinath Reddy Annapureddy, Sarath Kumar Bojedla, Hari D Yellamilli, Cheranjeevi Jayam

**Affiliations:** 1 Department of Transfusion Medicine and Hemotherapy, All India Institute of Medical Sciences, Mangalagiri, IND; 2 Department of Transfusion Medicine, Siddhartha Medical College, Vijayawada, IND; 3 Department of Orthopedics, All India Institute of Medical Sciences, Mangalagiri, IND; 4 Department of Nursing, All India Institute of Medical Sciences, Mangalagiri, IND; 5 Department of Radiology, All India Institute of Medical Sciences, Mangalagiri, IND; 6 Department of Nuclear Medicine, All India Institute of Medical Sciences, Mangalagiri, IND; 7 Department of Emergency Medicine, All India Institute of Medical Sciences, Mangalagiri, IND; 8 Department of Dentistry, All India Institute of Medical Sciences, Mangalagiri, IND

**Keywords:** hemolytic disease of the newborn, pemat score, patient education material, youtube videos, actionability, understandability

## Abstract

Introduction

With revolutions in Information Technology, information and misinformation are easier to be found online. YouTube is the largest and most commonly searched video content website in the world. It is assumed that, due to the coronavirus pandemic, most patients try to know about diseases through the internet and reduce the number of hospital exposures unless otherwise. In order to assess the understandability and actionability of such YouTube videos available freely online about the disease, Hemolytic disease of the newborn (HDN), this study was planned.

Methods

This is a cross-sectional study conducted with the first 160 videos available on May 14, 2021, with the search keyword "HDN" with a relevance filter and a duration of 4 to 20 minutes. The videos were further screened regarding the information content and language. These videos were assessed by three independent assessors using the patient educational materials assessment tool for audio-visual content.

Results

Out of the first 160 videos selected for screening, 58 videos were excluded due to a lack of content about the searched disease "HDN". Another 63 videos were excluded due to the language of instruction not being in English. Finally, 39 videos were assessed by three assessors. The understandability and actionability responses were checked for reliability and a Cronbach's alpha of 93.6% was found, indicating good data reliability. To reduce subjectivity, average scores of understandability and actionability were taken based on the scores of these three assessors. There were eight and 34 videos with average understandability and actionability scores of <70% respectively. The median average understandability and actionability scores were 84.4% and 50% respectively. There was a statistically significant difference between understandability and actionability scores with considerably lower actionability scores of YouTube videos on the disease, HDN (p<0.001).

Conclusion

There is a great need to include actionable information by content developers in videos. Most information available has adequate understandable content making it easier for the general public to know about the diseases. YouTube and similar social sites thus possibly are helping in the dissemination of information promoting awareness among the public in general and patients in particular.

## Introduction

With the advances in networking and faster speeds of communications, the internet has become the primary source of information for patients, patient groups, self-help groups, and educational and commercial stakeholders both to generate awareness as well as to help in terms of the next step to be taken by them in the course of disease management [1]. No author knowingly falsifies any information. However, sometimes, some of the information might not be true and may be perceived by the author to be accurate to the best of his knowledge. Sometimes the information may be correct but may be presented in a way that the reader or viewer may get a false notion due to the complexity of the presentation. Understanding health information and whether the information is accurate and actionable is an important task. Misinformation may decrease people’s quality of life and increase mortality risk [2].

One example of misinformation is a paper published on Measles, Mumps, and Rubella vaccination causing autism [3], refuted later by the scientific community [4], and the publication was eventually retracted. This incident was followed by evidence of multiple measles outbreaks locally [5,6] and the World Health Organization revoking the measles eradication status of four countries globally [7]. Whether the attributable cause is due to the negative impact of information socially is unknown. From this, it may be assumed that online information may have beneficial and harmful effects on the community.

Alloimmunization during pregnancy is a common occurrence that happens in about 1% of all pregnant women not receiving any intervention against the Rhesus (Rh) system alone causing Hemolytic disease of the newborn (HDN). The first known record of hydrops fetalis was by Hippocrates of a “fleshy fetus” with general “fetal dropsy” [8]. HDN is characterized by excessive hemolysis of fetal or neonatal red blood cells leading to fetal and/or neonatal anemia, jaundice, and/or erythroblastosis [9]. The common cause of the disease is the passive transfer of maternal alloantibodies through the placenta which may be directed against ABO antigens, Rh antigens, or other antigens present in the red cells [10,11]. Depending on the type of antibody and its specificity, the prognosis, severity, clinical manifestations, and treatment may vary [12].

This study has been planned to ascertain the usefulness of YouTube videos pertaining to HDN as patient education material in terms of understandability and actionability using the Patient Education Materials Assessment Tool-Audio/Visual (PEMAT-A/V) [13].

## Materials and methods

This study was planned as a cross-sectional, observational, study. A sample size of 30 videos was considered sufficient to provide adequate statistical data; however, keeping in view the videos in multiple languages and the possible exclusion of videos a total of the first 160 videos were considered for initial screening.

The first 160 YouTube videos, which were shown when the search term "HDN" was used with specific search parameters on May 14, 2021, were included in the present study.

Videos with content different from HDN and languages other than English were excluded from the study.

YouTube search parameters

The YouTube website was searched using the term “HDN” from its inception to May 2021. The selected video search and filter settings were based on relevance and video length of between 4 to 20 minutes. The first 160 videos were added to a new playlist.

Video exclusion assessment

Three independent assessors have watched these videos for qualification into the study pertaining to content and language. Videos meeting exclusion criteria were excluded. The excluded videos were removed from the playlist by an independent reviewer after verification again.

Video assessment process and blinding protocol

Three different assessors reviewed the videos left in the playlist for understandability and actionability assessment based on the PEMAT-AV [13]. The assessors included one each from the medical, paramedical, and nursing professions to ensure that they had some baseline understanding of the disease. The assessors were trained independently to assess videos without bias, to think from a patient perspective without any knowledge about the disease, and to give score points for each of the 17 minor assessment categories in the survey form. All three assessors have been given code names as "A", "i", and "1" respectively, and confidentially. The principal investigator alone knows the code names of all the individuals. The questions of the PEMAT-AV have been incorporated into a google survey form and distributed to the individual assessors for use in the assessment of videos at independent locations in order to avoid bias. The final database thus obtained has been checked for the completion of the survey by the independent reviewer in order to eliminate bias. The final database then was used for statistical analysis by the principal investigator.

Assessment tool

Although there are many tools that describe various parameters of audio-visual materials, the investigators have selected the PEMAT-AV [13] in view of the required parameter’s understandability and actionability and in view of its complexity. To eliminate subjective bias, averages of the individual scores of assessors were taken for further statistical analysis.

Assessment criteria under PEMAT-AV

There are a total of 17 assessment parameters (questions); 13 and four for understandability and actionability respectively with further sub-parameters (Table 1).

**Table 1:** PEMAT-A/V assessment variables PEMAT = patient educational materials assessment tool; A/V = audiovisual material

S.No	Nature	Type	Scoring variable
1	Understandability	Content	The material makes its purpose completely evident
2		Word Choice and Style	The material uses common, everyday language
3			Medical terms are used only to familiarize the audience with the terms. When used, medical terms are defined
4			The material uses the active voice
5		Organization	The material breaks or “chunks” information into short sections
6			The material’s sections have informative headers
7			The material presents information in a logical sequence
8			The material provides a summary
9		Layout and Design	The material uses visual cues (e.g., arrows, boxes, bullets, bold, larger font, highlighting) to draw attention to key points
10			Text on the screen is easy to read (A/V)
11			The material allows the user to hear the words clearly
12		Use of Visual Aids	The material uses illustrations and photographs that are clear and uncluttered
13			The material uses simple tables with short and clear row and column headings
14	Actionability	Actionability	The material clearly identifies at least one action the user can take
15			The material addresses the user directly when describing actions
16			The material breaks down any action into manageable, explicit steps
17			The material explains how to use charts, graphs, tables, or diagrams to take actions

Statistical analysis

The results of assessments of the individuals were recorded into an Excel sheet and were analyzed using Microsoft® Excel. Individual and average actionability and understandability scores were calculated. Descriptive statistics were performed using Microsoft® Excel. The reliability statistics were performed by calculating Cronbach’s Alpha in Microsoft Excel using the formula, α= Nc/[v+(N-1)c] where α is Cronbach's alpha, N is the number of items, v is the average variance, c is the average inter-item covariance among the items. The average scores of understandability and actionability were again compared using Wilcoxon signed rank test to identify any statistically significant difference in the mean scores of the two groups [14].

## Results

Out of the first 160 videos screened, videos not having content on the HDN were found to be 58 in number and excluded with 102 videos left. Similarly, videos with audio language other than English were found to be 63 in number and were excluded. Thus, 39 videos qualified to be included in the study (Table 2).

**Table 2:** List of final videos assessed under the study HDN = Hemolytic disease of the newborn; HD = high definition; Rh = Rhesus; USMLE = United States Medical Licensing Examination

S.No	Title of the video	Running time	Creator	Year of upload
1	ABO and Rh Incompatibility of the Newborn, HDN, and Erythroblastosis Fetalis. Explained	04:27	Alan Gibson	2018
2	ABO Incompatibility And Hemolytic Disease Of The Newborn (HDN)	13.58	Medicosis Perfectionalis	2018
3	Ch. 19, pt. 8 - Hemolytic Disease	05:56	Human Anatomy, Histology and Physiology	2021
4	Ch14 Rh INCOMPATIBILITY | BLOOD PHYSIOLOGY	10:23	Dr Hardik Mistry	2018
5	Clinical Correlation: Hemolytic Disease of the Newborn	09:10	Anatomy and Physiology for Paramedics	2015
6	Erythroblastosis Fetalis	16:29	CanadaQBank	2015
7	Erythroblastosis Fetalis	06:16	Olivia Kaminski	2020
8	Erythroblastosis Fetalis	06:40	Natalya Machado	2020
9	Erythroblastosis Fetalis (Hemolytic Disease of the Newborn)	17:30	MyelinNation	2018
10	Erythroblastosis fetalis | effects of erythroblastosis on newborn | Rh incompatibility in pregnancy |	10:16	Medico Stuff	2020
11	Erythroblastosis fetalis | Rh Incompatibility	05:04	Hussain Biology	2018
12	Erythroblastosis Fetalis or Hemolytic Disease of the Newborn or Rh Incompatibility	11:15	Now I Know	2019
13	ERYTHROBLASTOSIS FETALIS #csirgateicmr	11:58	The 24 Life sciences	2019
14	Group 4: Hemolytic Disease of Newborn	06:59	NUR SYAFIKA BINTI RAMLEE	2021
15	Haemolytic disease of the new born (Erythroblastosis Featalis) part 2	06:28	Sindy’s Tales	2019
16	Haemolytic Disease of the Newborn (Rhesus System) - Medical Tutorial	11:26	TachyTutorials	2020
17	HDN: Hemolytic Disease of the Newborn	04:10	NaickerN	2011
18	Hemolytic anemia of newborns	11:17	Joe DeMasi	2017
19	Hemolytic Disease of New Born Part 2 (HD)	15:31	Rabiul Haque	2017
20	Hemolytic disease of new born part-I	12:08	tutan medical class	2018
21	Hemolytic Disease of Newborn	08:18	Dr Dipti Thakker Kariya Physiology lectures	2018
22	Hemolytic disease of the newborn	14.54	Dr. John Campbell	2015
23	Hemolytic Disease of the Newborn	08:19	Physiology & Anatomy Videos	2014
24	Hemolytic Disease of the Newborn EXPLAINED | Rh and ABO incompatibility	09:36	Quick Med	2021
25	Hemolytic Disease of the Newborn HDN	05:01	Biology-Jose Maldonado EPCC	2020
26	Hemolytic disease of the newborn -or- erythroblastosis fetalis	07:14	Ren Hartung	2016
27	Hemolytic Disease of the Newborn, Animation	04:11	Alila Medical Media	2019
28	Hemolytic Disease of the newborn/ Rh Factor. (Chapter 19)	06:21	The Old Grace Is Dead	2014
29	Newborn Hemolytic Diseases (Heme/Onc) - USMLE Step 1	09:17	MadMedicine	2019
30	Pathophysiology of Hemolytic Disease of the Newborn	12:29	Health Ed Solutions	2012
31	RH blood group. RH Factor and Erythroblastosis	06:51	Meerab Fatima	2020
32	Rh Incompatibility | NCLEX REVIEW	08:26	That nursing prof	2021
33	Rh Incompatibility || HDN || Erythroblastosis Foetalis || In ENGLISH, Dr. Roopam Jain	15:51	Dr. Roopam Lectures	2019
34	Rh incompatibility and Hemolytic disease of the newborn (HDN)	17.49	Medicosis Perfectionalis	2018
35	Rh Incompatibility I Hemolytic Disease of the Newborn I Swatilekha Das	07:29	Swatilekha Das Nursing Classes	2018
36	Rh Incompatibility: Hemolytic Disease of the Newborn (Erythroblastosis fetalis) (FL-Immuno/65)	12:25	Frank Lectures	2017
37	RH-INCOMPATIBILITY (ERYTHROBLASTOSIS FETALIS)	09:31	Nursing notes	2020
38	What is erythroblastosis fetalis?	04:28	Biology with Shraddha	2020
39	What is Erythroblastosis Foetalis? Rh Incompatibility	07:11	HAPPY MIND HAPPY YOU	2019

The Cronbach’s Alpha was assessed for reliability before further testing of data. We found it to be 0.8651 (86.51%) indicating reliable data. This was followed by individual scoring of videos.

**Figure 1:**
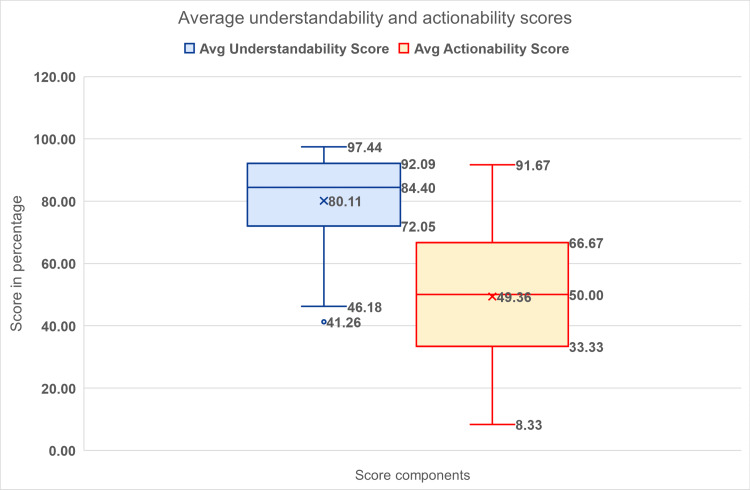
Understandability and actionability scores of individual assessors Avg = average

The understandability and actionability scores of individual assessors for the videos using the PEMAT-A/V tool [13], ranged from 7.69% to 100% and 0% to 100% respectively. The median scores were 84.62% and 50% respectively for understandability and actionability respectively (Figure 1).

**Figure 2:**
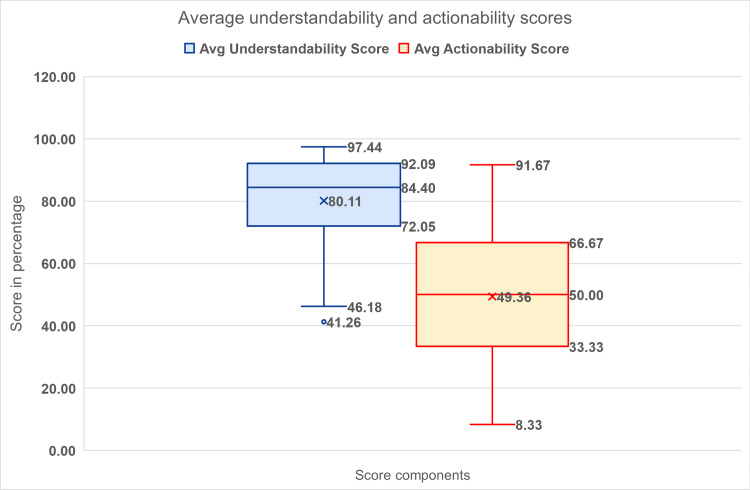
Average understandability and actionability scores of videos Avg = average

In order to reduce the subjective nature of individual assessors, the average understandability and actionability scores of three assessors were calculated for each video. These scores then ranged from 41.26% to 97.44% and 8.33% to 91.67% for understandability and actionability respectively (Figure 2). The median average of understandability and actionability scores were 84.4% and 50% respectively.

**Figure 3:**
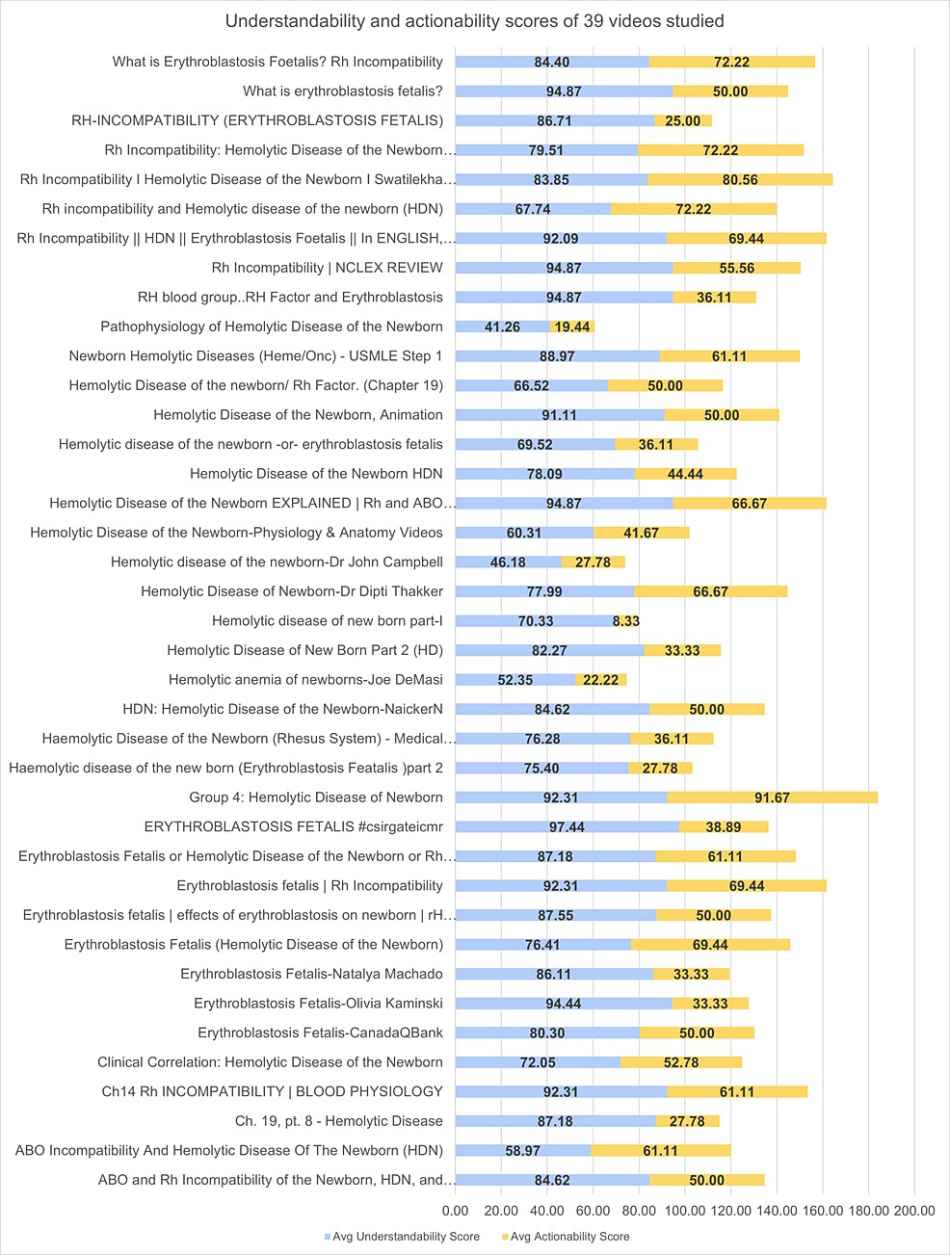
Average understandability and actionability scores of videos HDN = Hemolytic disease of the newborn; HD = high definition; Rh = Rhesus

The average understandability and actionability scores of 39 videos were depicted in Figure 3. The Wilcoxon signed-rank test [14] was performed to assess the presence of any statistically significant difference between the average understandability and actionability scores of the videos with a significance level of 0.05 and a two-tailed hypothesis with a final W-value of six, Z-value of -5.3587 with a p-value of <0.00001.

## Discussion

The credibility of source health information is important and is affected by the expertise (user-generated information, patients, health professionals, pharmaceutical companies, etc.) and trustworthiness of the information provider (Media, Scientific articles, Government guidelines, etc.). Online information is available in eBooks, ePapers, eMagazines, online radio, online television channels, pharmaceutical advisers, direct online sources, search engines, and mobile apps. Google has become the world’s most visited site followed by YouTube and YouTube is the second most visited social media platform and the world's first website with audiovisual content [15,16].

With the ongoing Corona viral pandemic, people are increasingly turning towards online options for understanding disease and consultations. The apprehension and fear of the pandemic have made people panic about going to a hospital because of assumed increased exposure to possible patients. YouTube [17] is one of the commonest search engines employed. Assuming the same, it is likely that patients try to seek help with information about HDN and what to do next if diagnosed with it. More and more videos creep up on YouTube daily from multiple sources and appraisal of such online videos for quality and safety is of utmost importance. Leave alone the accuracy, usability, and quality, in some instances the understandability and actionability of the videos will be poor.

There are multiple tools available online with the advent of the internet, used to assess various parameters for printed online education materials like the suitability assessment of materials tool [18], and DISCERN instrument [19]. With the incorporation of audio and video content into patient education materials, extra parameters were to be assessed and one such tool is the Patient Education Materials Assessment Tool created by the Agency for Healthcare Regency and Quality to assess the understandability and actionability of printed and audio-visual patient education materials.

In the present study, though the understandability and actionability scores of individual assessors had extreme values, on averaging the individual scores, the subjective variations could be removed. In the average scores, none of the videos got scores of either 0% or 100% indicating that all three assessors did not have similar opinions especially in assessing the extreme score videos, and not even a single video could elicit a score of 0% or 100% by all the three assessors.

There was a statistically significant difference between the understandability and actionability scores reflecting poor actionability. This can also be seen from the presence of only eight videos with average understandability of <70% while 34 videos had average actionability scores of <70%.

There seems to be an evolution of content providers in terms of understandability and actionability as may be evidenced by the presence of lower scores in the older videos than in the newer videos and studies. There are no studies on the assessment of understandability and actionability of YouTube videos on HDN, however, there are many studies on other diseases/conditions like online cardiovascular disease risk calculators [20], type 2 diabetic risk calculators [21], diabetes mellitus [22], laryngectomy [23], clubfoot [24], chest pain in children [25], etc., including one more similar study by author on Hemophilia [26]. Most studies had similar good understandability scores, but poor actionability scores.

The limitations of the study include the utilization of only medium-length videos for understandability and actionability assessment.

## Conclusions

To conclude, content developers are doing good service in providing essential information to the community but can further improve the understandability and actionability of YouTube videos of medium duration (4-20 minutes) on HDN to help patients get information. There is a scope for improvement in a video presentation to improve understandability as may be evidenced by the number of videos without relevant content found during the initial search. The video content should include more details on what to do next and where to go after diagnosis. More information may also be provided regarding disease modifiers and prevention strategies to give more actionable options for viewers.
